# XDR-TB in South Africa: No Time for Denial or Complacency

**DOI:** 10.1371/journal.pmed.0040050

**Published:** 2007-01-23

**Authors:** Jerome Amir Singh, Ross Upshur, Nesri Padayatchi

## Abstract

Singh and colleagues discuss the threat to regional and global public health posed by XDR-TB in KwaZulu-Natal, and propose new measures to control the outbreak.

On September 1, 2006, the World Health Organisation (WHO) announced that a deadly new strain of extensively drug-resistant tuberculosis (XDR-TB) had been detected in Tugela Ferry (Figure 1), a rural town in the South African province of KwaZulu-Natal (KZN) [1], the epicentre of South Africa's HIV/AIDS epidemic. Of the 544 patients studied in the area in 2005, 221 had multi-drug-resistant tuberculosis (MDR-TB), that is, Mycobacterium tuberculosis that is resistant to at least rifampicin and isoniazid. Of these 221 cases, 53 were identified as XDR-TB (see Table 1 and [2]), i.e., MDR-TB plus resistance to at least three of the six classes of second-line agents [3]. This reportedly represents almost one-sixth of all known XDR-TB cases reported worldwide [4]. Of the 53, 44 were tested for HIV and all were HIV infected.

**Figure 1:**
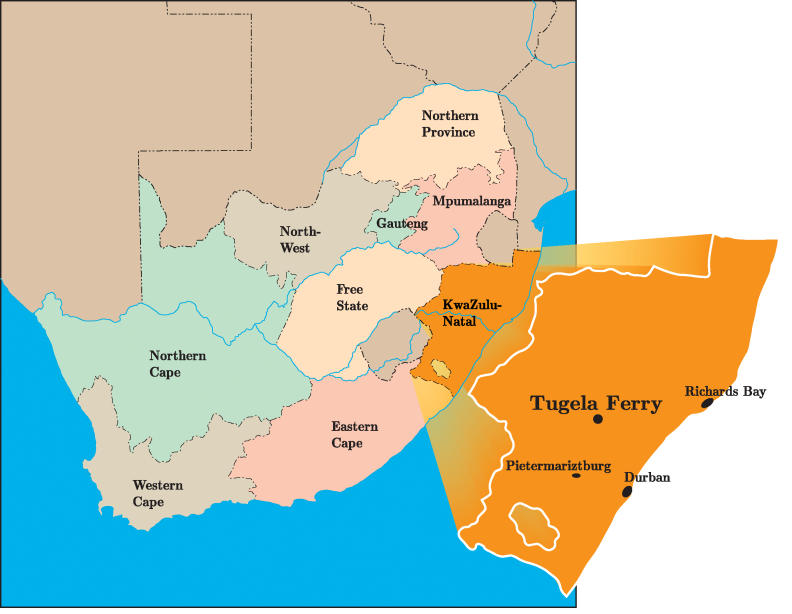
Map of South Africa Showing Tugela Ferry in the Province of KwaZulu-Natal, the Epicentre of South Africa's HIV/AIDS Epidemic

**Table 1:**
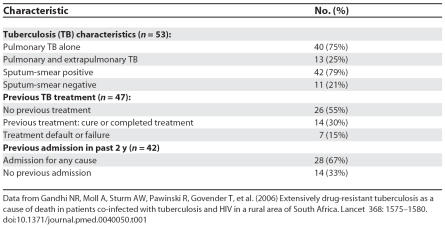
Characteristics of Patients in South Africa With XDR-TB

The median survival from the time of sputum specimen collection was 16 days for 52 of the 53 infected individuals, including six health workers and those reportedly taking antiretrovirals [2]. Such a fatality rate for XDR-TB, especially within such a relatively short period of time, is unprecedented anywhere in the world.

## The Threat to Regional and Global Health

South Africa is one of the world's fastest growing tourist destinations [5], home to millions of migrant labourers from neighbouring countries, and its ports and roads service several other African countries. Seroprevalence rates for HIV in South Africa, and in adjoining nations such as Lesotho and Swaziland, are very high. Cumulatively, these factors make for a potentially explosive international health crisis.

The threat to regional and global public health is thus clear [6], and further underlined by reports that XDR-TB is now considered endemic to KZN [7], as it has been reported in at least 39 hospitals throughout the province [8] and in other parts of the country [9–11]. At least 30 new cases of XDR-TB are reportedly detected each month in KZN alone [12].

## The True Extent of the Problem

Diagnosed cases of XDR-TB likely represent a small proportion of the true extent of the problem. The number of persons harbouring latent infections is unknown (and likely unknowable at present). Official statistics also likely underestimate the true prevalence of XDR-TB, as the current national TB guidelines prescribe the conditions under which M. tuberculosis susceptibility testing should be done [13]. These guidelines recommend susceptibility testing for those patients who have previously been treated for TB or fail to respond to treatment after two months of TB treatment, at which point there is a high treatment interruption rate. In addition, specialised laboratory facilities are required for such testing. Routine sputum culture and susceptibility testing of all patients suspected as having TB should form part of a multi-faceted approach to identifying and addressing TB drug resistance.

In recognition of the global threat posed by these factors, on September 9, 2006, WHO urged a response to the outbreak akin to recent global efforts to control severe acute respiratory syndrome (SARS) and bird flu [14]. The South African government's initial lethargic reaction to the crisis [15,16] and uncertainty amongst South African health professionals concerning the ethical, social, and human rights implications of effectively tackling this outbreak [17,18] highlight the urgent need to address these issues lest doubt and inaction spawn a full-blown XDR-TB epidemic in South Africa and beyond.

## Factors Fuelling the Outbreak

Several well-documented factors, including high treatment interruption rates of drug-sensitive TB and consequent low cure rates, together with the HIV epidemic, have contributed to the emergence of MDR-TB and XDR-TB in South Africa and merit urgent remediation. For instance, the development of drug resistance may result from inappropriate treatment regimens (e.g., choice of drugs, dosage, duration of treatment), programme factors (e.g., irregular drug supply, incompetent health personnel), and patient factors (e.g., poor adherence, mal-absorption). In fact, it could be said that the emergence of MDR-TB itself is evidence of the systematic failure of the global community to tackle a curable disease.

The factors that facilitate the spread of tuberculosis are well known and abundantly present in sub-Saharan Africa. Alongside inadequate health-care system response, poverty and global inequity contribute to the worsening of the global TB situation [19,20]. According to South Africa's Medical Research Council, about half of adults in South Africa with active TB are cured each year, compared with 80% in countries with better resources. Moreover, nationally, about 15% of patients default on the first-line six-month treatment, while almost a third of patients default on second-line treatment [21]. This highlights the urgent need for the health system (which includes health-care workers) to reinforce the DOTS (directly observed treatment, short-course) and DOTS-plus strategy, to revise current adherence counselling and public information strategies, and to actively promote avoidance of a “victim blaming approach”. The emergence of MDR-TB and XDR-TB is an indicator of the poor implementation of South Africa's TB Control Programme.

A neglected but significant factor fuelling the MDR-TB and XDR-TB outbreaks in South Africa [22] is the lack of infection control in institutions, including the lack of simple administrative measures such as triaging of patients, as well as more sophisticated expensive environmental control measures, such as negative pressure rooms and personal respiratory protection (respirators). Infection control must be addressed in order to reduce the nosocomial transmission of these infections.

In the modern era, tuberculosis is recognised as a disease that preys upon social disadvantage [23,24]. Thus, the inadvertent deterrent impact that health and social welfare policies are having on the hospitalisation of such patients needs to be explored. Currently, 10 million South Africans—almost one in four citizens—are beneficiaries of some form of social welfare [25]. With unemployment in South Africa conservatively estimated at about 27% of the population [26], social welfare grants often constitute the sole or primary income of many households. While South Africa does not have a formal universal health-care system, those who require but who cannot afford to pay for hospitalisation are often treated free of charge in the public sector [27]. However, current government policy stipulates that those who are hospitalised at state expense lose their social welfare benefits for the duration of their hospitalisation.

Faced with the prospect of being deprived of their gainful employment and/or having their welfare benefits suspended for the duration of hospitalisation—which in the case of MDR-TB or XDR-TB could last 18–24 months—many MDR-TB patients opt not to stay in hospitals, where their treatment adherence and resistance profile could be closely monitored by health personnel. Instead, understandably, these highly infectious individuals fail to receive appropriate therapy and are likely to default on adherence. They mix broadly in society among non-infected individuals, typically utilise public transport, and seek or continue their gainful employment. In so doing, they pose a significant public health risk to their families, co-workers, local community, and the wider public they encounter.

Given the cost of trying to manage a MDR-TB or XDR-TB epidemic [28], the South African government ought to rethink its policy of suspending welfare benefits to patients with MDR-TB or XDR-TB for the duration of their hospitalisation. Moreover, it ought to consider extending welfare benefits to those infected patients who are gainfully employed as an incentive to draw such patients into the health system so that their adherence to anti-TB medication and resistance profile can be monitored. Although these measures will undoubtedly have cost implications for the government and may not adequately compensate patients for their lost income, they would at least serve as some form of incentive and encouragement for infected individuals to enter and remain in the health system, although admittedly, their confinement could conceivably be indefinite or until they die. It would also be a partial realisation of the reciprocity principle, which we explore below.

## Factors that Could Undermine Efforts to Tackle the Outbreak

Several factors threaten to stymie efforts to control the XDR-TB outbreak in South Africa. Drug resistance can only be detected if a patient presents to the health system, a health-care worker suspects TB, an appropriate specimen is taken, facilities exist for smear and cultures, and if laboratories are equipped to do drug susceptibility testing. Moreover, because most hospital beds in South Africa are occupied by patients infected with opportunistic infections associated with HIV/AIDS, there is little or no spare capacity to accommodate patients with MDR-TB and XDR-TB. However, given the airborne transmission of TB and the grave threat that MDR-TB and XDR-TB pose immediately to such immunocompromised patients and, if the spread of XDR-TB is not abated, to global health, the government ought to reconsider its prioritisation of hospital resources. It seems that, at minimum, patients with XDR-TB requiring inpatient care should be housed in facilities independent both of patients with MDR-TB and patients who are immunocompromised. The containment of infectious patients with XDR-TB may arguably take precedence over any other patients not infected with highly infectious and deadly airborne diseases, including those with full-blown AIDS. This is an issue requiring urgent attention from the global community.

### Is There a Role for Involuntary Detention?

The successful containment of TB, MDR-TB, and XDR-TB in South Africa and elsewhere carries human rights [29] and ethical implications. An important question that we must come to terms with is the extent to which judicially sanctioned restrictive measures should be employed to bring about control of what could develop into a lethal global pandemic.

As diagnosis of MDR-TB and XDR-TB can take several weeks, questions remain about what to do with patients suspected of being infected with MDR-TB or XDR-TB while awaiting susceptibility results. And once patients have been determined to be infected, there are questions about how long and how closely their clinical status should be monitored and under what conditions. Ideally, patients suspected of having TB should be isolated in an acute infectious diseases setting while awaiting anti-tuberculosis drug-susceptibility testing, and then triaged for further management based on these results. Current WHO guidelines recognise that this strategy is not feasible in resource-constrained environments. WHO recommends that persons with MDR-TB voluntarily refrain from mixing with the general public and from those susceptible to infection, while they are infectious and in ambulatory care [30]. The document is silent on what steps to take should such voluntary measures fail.

The emergence of XDR-TB indicates that the WHO strategy of allowing the patient to assume responsibility for mixing with the general public may be too permissive and more attention to strategies of infection control in the community is required. In general, from both an ethical and legal perspective, measures that rely on voluntary cooperation and are the least restrictive in terms of interfering with human rights are preferred. However, if such measures prove to be ineffective, then more restrictive measures may need to be contemplated. Such measures should be taken with due consideration for the possibility that they may increase disincentives to seek care. However, if due care is taken to provide for the rights and needs of those so detained and therapeutic goals are kept paramount, such measures could play an important role in containing XDR-TB before it spreads more generally in the population globally.

Rights can usually be restricted if doing so is reasonable and justifiable.

The use of involuntary detention may legitimately be countenanced as a means to assure isolation and prevent infected individuals possibly spreading infection to others. However, South African officials have raised human rights concerns in dealing with the country's XDR-TB and MDR-TB outbreaks [18], although they have conceded that forcible treatment may be a viable option in tackling the outbreak [31]. Health workers and human rights advocates in South Africa and elsewhere must be reminded that although a country's Bill of Rights may bestow a range of human rights on individuals, these rights can usually be restricted if doing so is reasonable and justifiable. They should be made aware of any national laws and municipal by-laws that permit the provision of involuntary treatment and isolation measures in the interests of public health.

Moreover, the judiciary often has the authority to issue orders compelling involuntary confinement/hospitalisation and treatment, even against the wishes of an affected party, if doing so is in the public interest. This option should only be invoked if non-coercive measures have failed. Such an approach has been endorsed by the European Court on Human Rights (ECHR) in *Enhorn v. Sweden* [32]. The applicant in this case was an HIV-infected man who had infected another party and disobeyed the instructions of public health officials to desist from irresponsible and risky behaviour. The man complained to the ECHR that the compulsory isolation orders and his involuntary detention in a hospital had been in breach of Article 5(1) of the European Convention for the Protection of Human Rights and Fundamental Freedoms. This article states that “Everyone has the right to liberty and security of the person. No one shall be deprived of his liberty save in the following cases and in accordance with a procedure prescribed by law”. Section 5(1)(e) provides for the situation at hand: “the lawful detention of persons for the prevention of the spreading of infectious diseases”.

The applicant argued that the substantive provisions of Article 5 were not made out in his case, given that the detention did not constitute a proportionate response to the need to prevent the spread of infectious disease. The court held that any such detention must be in compliance with both the principle of proportionality and the requirement that there be an “absence of arbitrariness” such that other less severe measures have been considered and found to be insufficient to safeguard the individual and the public. This would entail that the deprivation of liberty was necessary in all circumstances [33].

Moreover, for detention to comply with principles of proportionality and freedom from arbitrariness, it must be established that the detained person is suffering from an infectious disease, that the spread of disease is dangerous to public safety, and that the detention of the infected person is the last resort measure in order to prevent disease spread. The court ruled that the institution of detention for infectious disease must be appropriate to the nature of the disease. Where these conditions are satisfied, deprivation of liberty is justified, both on grounds of public policy and in order to provide medical treatment to the affected party. In ruling in favour of the applicant the court found that the compulsory isolation of the applicant by Swedish authorities ought to have been considered only as a last resort in order to prevent him from spreading HIV after less severe measures had been considered and found to be insufficient to safeguard the public interest. We believe that the forced isolation and confinement of individuals infected with XDR-TB and selected MDR-TB may be an appropriate and proportionate response in defined situations, given the extreme risk posed by both strains and the fact that less severe measures may be insufficient to safeguard public interest. Patients with XDR-TB should also be quarantined separately from those with MDR-TB, as the latter is potentially curable.

Although the justness and effectiveness of forcibly confining and treating patients with TB [34,35] has been called into question [36], such an approach has met with some degree of success in the US [37], where it helped bring down TB infection rates in states such as New York in the 1990s [38]. We would not argue for forcible treatment of patients with MDR-TB or XDR-TB, simply restriction of mobility rights of such individuals.

Emulation of New York's aforementioned successful approach in controlling its TB outbreak could empower health officials in South Africa and elsewhere to act decisively in tackling emerging XDR-TB and MDR-TB outbreaks. The consequences of not educating health workers of the state's powers in such instances were highlighted on September 12, 2006, in Johannesburg, Africa's commercial and air transport hub, when health workers allowed a patient diagnosed with XDR-TB, who refused to be hospitalised, to discharge herself. Although this patient was eventually traced and forcibly hospitalised five days after her self-discharge [39], it remains unknown how many people she may have infected in the months between her sputum sample being taken and her eventual diagnosis in September 2006, and before she was traced after her self-discharge.

Questions also remain about how authorities should deal with patients with MDR-TB whom treatment has failed to cure as well as patients with XDR-TB in whom cure is unlikely as few active drugs remain. While isolating such patients until they die—which in the case of the slightly less deadly MDR-TB could be years—has been described as “ethically questionable and impractical” [21], this option may, of necessity, need to be countenanced. It is not, *a priori*, unethical to restrict the movement of those whose infection poses risks to public health. It is a matter of what types of safeguards are put in place to assure the legitimacy of such acts.

There are many such justifications emerging in the field of public health ethics that recognise that prevention of harm and protection of public health are legitimate ethical norms [40–42]. Human rights doctrine also recognises the limitation of many rights in a public health emergency, provided the measures employed are legitimate, non-arbitrary, publicly rendered, and necessary. In this regard, section 25 of the Siracusa Principles on the Limitation and Derogation of Provisions in the International Covenant on Civil and Political Rights holds: “Public health may be invoked as a ground for limiting certain rights in order to allow a state to take measures dealing with a serious threat to the health of the population or individual members of the population. These measures must be specifically aimed at preventing disease or injury or providing care for the sick and injured” [43]. It must be assured that detained individuals have appropriate legal council, and given the uncertainty of the duration of restrictions required, duly constituted independent tribunals could be established to oversee the process. At issue from a human rights perspective is whether such prolonged isolation represents the least restrictive means to achieve this goal and the extent of the belief in the severity of the threat. We do not intend to resolve this issue presently, but believe it is worth tabling for broader debate.

Human rights doctrine recognises the limitation of many rights in a public health emergency.

The use of legally sanctioned restrictive measures for the control of XDR-TB should not obscure the fact that being infected is not a crime. A strong reciprocal obligation is borne by authorities so wishing to invoke these measures. Those who are isolated require humane and decent living conditions. In fact the restriction of their liberties is more for a collective good than for their own. Thus every effort must be made to ensure conditions of living that preserve dignity. Harris and Holm have argued that all people with a communicable disease have a duty not to infect others. They stress, however, that “[i]t is...also a duty which we can expect people to discharge *only if they live in a community that does not leave them with all the burdens involved in discharging this duty*” [38] (italics ours). The task of global health is to help create these communities.

## Conclusion

XDR-TB is a serious global health threat. It has the potential to derail the global efforts to contain HIV/AIDS, as broadly disseminated XDR-TB will prove to be a much more serious public health threat owing to its mode of transmission. The emergence of XDR-TB is also an uncomfortable reminder of the failure of health systems to control problems at a tractable scale. If, in the recent past, TB were to have been adequately managed when it was completely drug sensitive, we would not be in such a dire situation as is currently the case. This failure rests upon us all. We should begin to contemplate the response when we move to the predictable next phase: completely drug-resistant tuberculosis.

By December 1, 2006—World AIDS Day—South Africa had reported more than 300 cases of XDR-TB [44] (based on the latest definition of XDR-TB, i.e., resistance to at least rifampicin and isoniazid, with resistance to one of the injectable drugs [kanamycin, amikacin, capreomycin] and one of the quinolones). Given the South African government's poor track record in dealing with the country's HIV/AIDS epidemic and what is at stake if it adopts a similar lethargic and denialist response to the country's XDR-TB outbreak, the international community must be vigilant in monitoring the government's response to this emerging crisis. Although recent initiatives of the government [45,46] and the Medical Research Council of South Africa [28,47] are encouraging, these will hopefully not inspire complacency amongst officials.

While it is encouraging that the South African government invited the WHO to an October 2006 meeting on the emerging crisis [48], it is worth noting that neither party raised the human rights and ethical dimensions of controlling the outbreak. Containing XDR-TB and selected MDR-TB will require an interdisciplinary approach [49] and the synergistic cooperation of all organs of the state, including, in particular, the judiciary, as well as various government departments. Moreover, the government should urgently consider devising strategies to control the disease amongst particularly high-risk groups such as prisoners and migrant labourers, which might necessitate the involvement of prisoner advocacy groups and neighbouring countries, respectively.

If WHO is sincere in calling for the XDR-TB outbreak in South Africa to be treated in the same light as SARS and bird flu, then global efforts to develop rapid diagnostic tests and novel treatment regimens must be stepped up. In addition to drug development, the appropriateness of using these technologies in countries with TB/HIV epidemics needs to be explored. The determination of XDR-TB requires specialised laboratories and quality assurance, particularly when testing for resistance to second-line anti-tuberculosis agents. Moreover, while the diagnosis of MDR-TB may take weeks or months, new technologies, including liquid culture and PCR probes, can reduce this time. Efforts must be stepped up to sponsor and equip poor countries to address these challenges. Depending on how successfully the South African government controls the outbreak, as in the case of SARS, infection monitoring at hospitals, border posts, and airports may become necessary.

Given the ethical and legal implications of these measures, the experience of countries that were affected by SARS [50] could prove valuable in guiding South Africa to deal with its XDR-TB outbreak. Admittedly though, more is known of XDR-TB than was the case with SARS when it first emerged. In the meantime, South Africa must urgently reduce crowding in hospitals where patients with TB are being treated to reduce the risk of the infection spreading, drastically expand its surveillance of the disease, and rethink its current counselling, treatment, reporting, and tracing strategies. It must also devise measures to reduce contact between patients with TB and those suspected or confirmed with MDR-TB and XDR-TB in the weeks or months it takes to diagnose the latter two infections. It must also devise appropriate infection-prevention strategies for health workers treating such patients.

All reasonable attempts must be made to accommodate the interests of infected patients in a sensitive and humane manner, although, if necessary, the government must adopt a more robust approach towards uncooperative patients with MDR-TB and XDR-TB, which might necessitate favouring the interests of the wider public over that of the patient. Although such an approach might interfere with the patient's right to autonomy and will undoubtedly have human rights implications, such measures are reasonable and justifiable, and must be seen in a utilitarian perspective. Ultimately in such crises, the interests of public health must prevail over the rights of the individual.

## 

**Figure:**
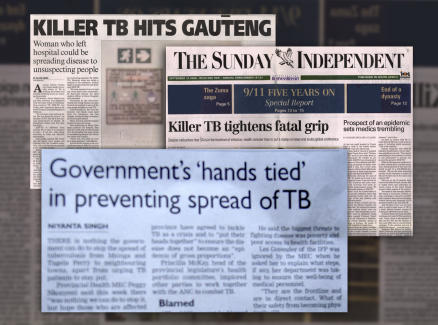
South African newspapers fuelled alarm about XDR-TB (figure: Anthony Flores)

## Abbreviations

ECHR: European Court on Human Rights
KZN: KwaZulu-Natal
MDR-TB: multi-drug-resistant tuberculosis
SARS: severe acute respiratory syndrome
TB: tuberculosis
WHO: World Health Organization
XDR-TB: extensively drug-resistant tuberculosis
